# Association of *Helicobacter pylori* with Serum HIF-1α, HIF-2α, and Human Transmembrane Prolyl 4-Hydroxylase Activity in Patients with Chronic Gastritis

**DOI:** 10.3390/medicina61071174

**Published:** 2025-06-28

**Authors:** Sefa Ergün, Fadime Kutluk, Basar Can Turgut, Seyma Dumur, Uğurcan Sayılı, Dilek Duzgun Ergun, Hafize Uzun

**Affiliations:** 1 Department of General Surgery, Cerrahpaşa Faculty of Medicine, Istanbul University-Cerrahpasa, Istanbul 34320, Turkey; 2 Department of General Surgery, Istanbul Avcılar Murat Kölük State Hospital, Istanbul 34320, Turkey; 3 Department of General Surgery, Binali Yildirim University Mengücek Gazi Training and Research Hospital, Erzincan 24100, Turkey; drkutluk@hotmail.com; 4 Department of General Surgery, Istanbul Training and Research Hospital, University of Health Sciences, Istanbul 34098, Turkey; basarcanturgut@gmail.com; 5 Department of Medical Biochemistry, Faculty of Medicine, İstanbul Atlas University, Istanbul 34403, Turkey; seyma_dumur@hotmail.com (S.D.); huzun59@hotmail.com (H.U.); 6 Department of Public Health, Cerrahpasa Faculty of Medicine, Istanbul University-Cerrahpasa, Istanbul 34320, Turkey; ugurcan.sayili@iuc.edu.tr; 7 Department of Biophysics, Faculty of Medicine, Istanbul Aydin University, Istanbul 34513, Turkey; dilekergun@aydin.edu.tr

**Keywords:** gastritis, *Helicobacter pylori*, hypoxia-inducible factor-1α, HIF-2α, lipid profile, transmembrane prolyl 4-hydroxylase

## Abstract

*Background and Objectives*: Chronic mucosal infection with *Helicobacter pylori* (*H. pylori*) plays a key role in the development of gastroduodenal disorders such as chronic gastritis, peptic ulcers, gastric lymphoma, and gastric cancer by triggering local immune responses and inducing hypoxic and inflammatory conditions in the gastric mucosa. This study aims to evaluate the potential diagnostic value of hypoxia-inducible factors HIF-1α and HIF-2α, along with transmembrane prolyl 4-hydroxylase (P4H-TM), as biomarkers in *H. pylori*-positive patients. Additionally, the study investigates the association between these markers and alterations in lipid profiles, as well as their involvement in the molecular mechanisms underlying gastric conditions like gastritis, particularly in the context of H. pylori infection. *Materials and Methods*: This study was conducted at Istanbul Avcılar Murat Kölük State Hospital’s General Surgery Outpatient Clinic. A total of 60 participants were included: 40 patients diagnosed with chronic gastritis (20 *H. pylori*-positive and 20 *H. pylori*-negative) and 20 healthy controls confirmed negative by 13C-urea breath test. Blood samples were collected for ELISA analysis of HIF-1α, HIF-2α, and P4H-TM levels. Additionally, lipid profiles were measured and compared among the groups. *Results:* No significant differences were found among the groups in terms of demographic factors such as age, sex, or body mass index (BMI). However, significant variations were observed in the levels of HIF-1α, HIF-2α, and P4H-TM across all groups (*p* < 0.001 for each marker). These markers were substantially elevated in the *H. pylori*-positive gastritis group compared to both the H. pylori-negative and healthy control groups. Receiver Operating Characteristic (ROC) curve analysis revealed that all evaluated markers exhibited strong diagnostic accuracy in differentiating H. pylori-positive individuals from other groups. HIF-1α (AUC: 0.983) and HIF-2α (AUC: 0.981) both achieved 100% sensitivity with specificities of 93.3% and 91.1%, respectively. P4H-TM showed an AUC of 0.927, with 85% sensitivity and 95.6% specificity. *Conclusions:* These findings indicate that HIF-1α, HIF-2α, and P4H-TM may serve as effective biomarkers for diagnosing *H. pylori*-positive patients and may be linked to changes in lipid metabolism. The elevated expression of these markers in response to *H. pylori* infection highlights their potential roles in the inflammatory and hypoxic pathways that contribute to the pathogenesis of gastric diseases such as gastritis.

## 1. Introduction

Chronic gastritis (CG) refers to the long-standing inflammation of the stomach lining, which can arise from a variety of causes, including infections, irritants, or autoimmune responses. Over time, this condition can result in damage to the stomach lining, impairing its ability to produce protective mucus against harsh acidic digestive juices. *Helicobacter pylori* (*H. pylori*) is a helical, microaerophilic, gram-negative bacterium that is classified as a class I human carcinogen. It is the most prevalent cause of chronic infection in humans. The bacterium colonizes the gastric mucosa due to its specialized motility, ability to thrive in low-oxygen environments, and capacity to neutralize stomach acid. Once colonized, *H. pylori* induce chronic inflammation of the gastric mucosa, employing several mechanisms to evade the host’s immune defenses. Chronic infection with *H. pylori* plays a crucial role in the development of gastroduodenal diseases, including chronic gastritis, peptic ulcers, gastric lymphoma, and gastric cancer, through local immune responses. *H. pylori* infection is a major risk factor for gastric cancer development. It induces chronic inflammation and gastric mucosal damage, leading to genetic and epigenetic changes that promote carcinogenesis. Environmental and genetic factors influence the persistence and pathogenicity of *H. pylori* infection. Environmental factors such as diet, smoking, and hygiene affect bacterial colonization and host response, while genetic variations in both the host and bacterium modulate immune evasion and disease severity, complicating treatment and management [1,2].

The primary protein involved in regulating the cellular response to hypoxia is the hypoxia-inducible factor (HIF). This family of transcription factors supports both oxygen transport and cellular adaptation under low oxygen conditions by controlling the expression of genes linked to crucial biological pathways such as glucose metabolism, erythropoiesis, angiogenesis, cell proliferation, and apoptosis. In humans, three distinct HIF isoforms—HIF-1, HIF-2, and HIF-3—have been characterized in response to hypoxic environments. These function as heterodimers composed of an oxygen-sensitive α subunit and a stable β subunit, which dissociate under normal oxygen levels. Despite sharing substantial sequence homology and overlapping target genes, HIF-1α and HIF-2α regulate distinct gene networks and exhibit different expression patterns. While HIF-1α is expressed across nearly all cell types, HIF-2α expression is limited to select cells and certain tumors. HIF-1α predominantly mediates the cellular response to short-term (acute) hypoxia, whereas HIF-2α is more critical in the response to prolonged (chronic) oxygen deprivation. HIF-1α mainly regulates glycolysis and acute hypoxia responses, while HIF-2α controls genes related to erythropoiesis and long-term adaptation. Their gene targets and expression patterns differ. HIF-1α is rapidly induced in acute hypoxia and promotes glycolysis and survival pathways, while HIF-2α shows sustained expression in chronic hypoxia, regulating angiogenesis, erythropoiesis, and long-term adaptation [3,4]. Furthermore, studies have shown that myeloid-derived HIF-1α has a protective effect on *H. pylori*-induced gastritis, emphasizing the complex interaction between innate immunity and inflammation in the development of gastric pathology [5].

Transmembrane prolyl 4-hydroxylase (P4H-TM) is an enzyme that is involved in the post-translational modification of proteins. It specifically catalyzes the hydroxylation of proline residues in proteins, which is crucial for stabilizing collagen and other extracellular matrix proteins. P4H-TM is a type of prolyl 4-hydroxylase that spans the membrane of the endoplasmic reticulum, which is essential for proper protein folding and maintaining the structural integrity of various tissues. The activity of P4H-TM is especially important in processes like wound healing, fibrosis, and collagen biosynthesis [6,7]. Recombinant P4H-TM hydroxylates two critical proline residues in the oxygen-dependent degradation domains of HIF-1α, preferentially targeting the C-terminal prolines in vitro, while it does not hydroxylate any prolines in recombinant type I procollagen chains. Under normoxic conditions, it contributes to protein folding and collagen biosynthesis by hydroxylating specific proline residues in target proteins within the endoplasmic reticulum. Its activity is influenced by hypoxia, during which altered hydroxylation patterns may affect protein stability and extracellular matrix integrity [8]. HIF-1α activation modulates the host response to bacterial infections by enhancing the transcription of genes involved in innate immunity, such as those encoding antimicrobial peptides, pro-inflammatory cytokines (e.g., IL-1β, TNF-α), and glycolytic enzymes. This promotes macrophage activation, supports metabolic adaptation under hypoxia, and boosts phagocytic and bactericidal activity, thereby strengthening the immune defense [7].

Recent research highlights the pivotal role of HIF-1α in the context of bacterial infections. Its activation appears to be influenced by both the type of invading pathogen and host-specific factors related to the infection. However, it is still not fully understood whether the induction of HIF-1α during infection confers a beneficial protective response or contributes to disease progression and host damage [9]. The aim of this study is to investigate the potential role of HIF-1α, HIF-2α, and P4H-TM as biomarkers in diagnosing *H. pylori* (+) patients and their association with lipid profile changes. Additionally, the study aims to explore the involvement of these biomarkers in the molecular mechanisms underlying gastric diseases like gastritis, particularly in response to *H. pylori* infection, which induces inflammatory and hypoxic responses in the gastric mucosa. Recognizing this relationship may be vital for initiating timely treatment and achieving effective elimination of *H. pylori*, which could substantially contribute to reducing the risk of developing gastric cancer.

## 2. Material and Methods

Prior to participation, all subjects provided written informed consent. The study protocol received ethical approval from the Ethics Committee of Istanbul University-Cerrahpasa, Cerrahpasa Medical Faculty (Approval No.: E-83045809-804.01-1210702; Date: 24 December 2024). All procedures were carried out in compliance with the ethical standards outlined in the Declaration of Helsinki.

This prospective observational study was carried out at the General Surgery Outpatient Clinic of Istanbul Avcılar Murat Kölük State Hospital. A total of 60 participants were enrolled, including 40 patients diagnosed with CG based on histopathological evaluation: 20 patients with *H. pylori*-positive gastritis and 20 patients with H. pylori-negative gastritis. The control group consisted of 20 healthy volunteers who confirmed that they were negative for H. pylori by the *13C-urea breath test (13C-UBT)*.

### 2.1. Inclusion Criteria

Patients who were over 18 years old and had complaints of epigastric pain and dyspepsia underwent upper gastrointestinal endoscopy, and biopsy results from their stomach antrum were evaluated. Patients diagnosed with chronic gastritis were categorized into two groups based on *H. pylori*-positive and *H. pylori*-negative, as confirmed by pathological evaluation and 13C-UBT.

A priori power analysis was conducted to ensure that the sample size was sufficient to detect statistically significant differences in the expression levels of HIF-1α, HIF-2α, and P4H-TM between groups. The power analysis indicated that a total sample size of 60 patients would provide over 80% power to detect medium to large effect sizes at a significance level of 0.05.

### 2.2. Exclusion Criteria

Exclusion criteria comprised individuals under 18 years of age; patients with psychiatric disorders, including psychotic or bipolar conditions; those with eating disorders; pregnant or lactating women; individuals with known malignancies or existing gastric or hepatic diseases; users of vitamin supplements or anticoagulant therapy; patients receiving high-dose corticosteroids; those with chronic or metabolic diseases; and participants unwilling to provide informed consent or with incomplete clinical or laboratory data. Patients with known autoimmune diseases or other significant comorbid clinical conditions were excluded during the selection process.

### 2.3. Sample Collection and Processing

Fasting venous blood samples were collected from all participants between 08:00 and 10:00 AM to control circadian variations. Blood was allowed to clot at room temperature for 30 min, then centrifuged at 1000× *g* for 20 min at 4 °C. The serum was carefully separated to avoid hemolysis, aliquoted into pre-labeled cryovials, and stored at −80 °C until analysis. Samples showing hemolysis, icterus, or lipemia (HIL) were excluded to ensure assay accuracy.

### 2.4. Measurement of Biomarkers

Serum concentrations of hypoxia-inducible factors HIF-1α, HIF-2α, and transmembrane prolyl 4-hydroxylase (P4H-TM) were quantified using commercially available sandwich ELISA kits (Thermo Fisher Scientific, Waltham, MA, USA) according to the manufacturer’s instructions (Human HIF-1α: Cat. No. E0422Hu; HIF-2α: Cat. No. E7164Hu; P4H-TM: Cat. No. E7241Hu; all provided by BT LAB, Jiaxing, China). Serum levels of target proteins were measured using commercially available ELISA kits according to the manufacturer’s instructions. Briefly, samples and standards were added to pre-coated plates, incubated, washed, and then treated with enzyme-linked antibodies. The colorimetric reaction was developed and quantified using a microplate reader at the specified wavelength. All assays demonstrated intra-assay and inter-assay coefficients of variation below 10%, confirming reliability and reproducibility.

### 2.5. Biochemical Analysis

Biochemical analyses were conducted via spectrophotometric methods using an automated analyzer (Hitachi Modular System, Roche Diagnostics, Hague Road, Indianapolis, IN, USA). C-reactive protein (CRP) levels were assessed by turbidimetric measurement using the ADVIA 1800 Auto Analyzer (Siemens Medical Solutions, Deerfield, IL, USA). Erythrocyte Sedimentation Rate (ESR) was measured to assess systemic inflammation.

### 2.6. 13C-Urea Breath Test

The urea breath test is a non-invasive, simple, and safe test used to diagnose H. pylori infection and to show its eradication after treatment. To select the negative group for Helicobacter infection, blood was taken from asymptomatic patients who did not receive proton pump inhibitor therapy or antibiotic therapy and had negative urea breath test results. The 13C-UBT was performed on all participants using the Refex test meal on day 30. Baseline breath samples were collected before ingestion of 200 mL of water with the Refex meal, followed by 75 mg of 13C-urea dissolved in 30 mL of water. A second breath sample was collected 30 min post-ingestion. A delta value ≥ 2.5‰ was considered positive for H. pylori infection [10].

### 2.7. Statistical Analysis

Statistical analysis was carried out using IBM SPSS Statistics version 21.0 (IBM Corp., Armonk, NY, USA), while graphical data visualizations were created using JASP version 0.18.3.0 and Jamovi version 2.4.11. Categorical data were summarized as counts and percentages, whereas continuous variables were expressed either as mean ± standard deviation (SD) or as median with interquartile range (IQR), depending on the distribution characteristics. The Shapiro–Wilk test was applied to evaluate the normality of continuous data. To assess differences in categorical variables across study groups, the chi-square test was used. Depending on the distribution, comparisons of continuous variables among three or more groups were conducted using either one-way ANOVA (for normally distributed data) or the Kruskal–Wallis test (for non-normally distributed data). In cases where overall group differences were significant, post hoc analyses were performed using Tukey’s test or adjusted *p*-values to identify pairwise differences. For comparisons between two independent groups, the independent samples *t*-test was applied to data with a normal distribution, while the Mann–Whitney U test was used for skewed data. For within-group comparisons involving paired data, either the paired *t*-test or Wilcoxon signed-rank test was employed, based on data normality. To evaluate the diagnostic accuracy of HIF-1, HIF-2, and P4H-TM, ROC curve analyses were conducted. The AUC, as well as sensitivity, specificity, and optimal cutoff values, was reported. Cutoff points were derived using the Youden index (J = Sensitivity + Specificity − 1) and rounded to the nearest clinically practical value to balance diagnostic efficiency and usability. Sensitivity is the ability of a diagnostic test to correctly identify individuals who have the condition of interest. It is defined as the ratio of true positives to the sum of true positives and false negatives, mathematically expressed as sensitivity = true positives/(true positives + false negatives). Specificity is the test’s ability to correctly identify individuals who do not have the condition. It is calculated as the ratio of true negatives to the sum of true negatives and false positives, that is, specificity = true negatives/(true negatives + false positives). A significance threshold of *p* < 0.05 was adopted for all statistical tests.

## 3. Results

No significant differences were observed in the proportions across groups. The percentage of females was 50% in the CG/HP (+) group, 55% in the CG/HP (−) group, and 52% in the control group. Demographic factors such as age (*p* = 0.995) and BMI (*p* = 0.675) did not show any significant variation across groups, indicating that the study populations were well-matched (Table 1). Routine laboratory parameters, including RBC, HGB, PLT, ESR, and CRP, did not differ significantly between the groups (*p* > 0.05 for all). However, notable differences were found in WBC counts (*p* = 0.031), with the control group showing the highest values. Additionally, lipid profile parameters, including cholesterol (*p* = 0.002), triglycerides (*p* = 0.018), and LDL (*p* < 0.001), were significantly higher in the CG/HP (+) group (Table 1).

**Figure 1:**
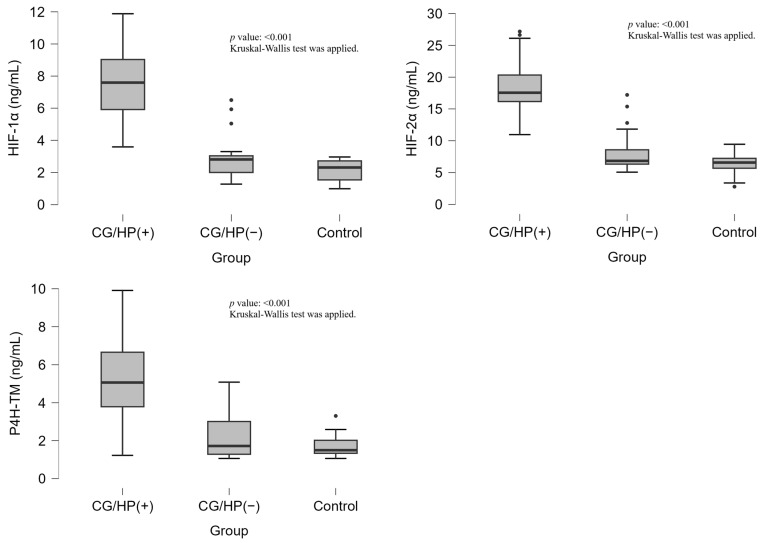
Box-plot Distribution of HIF-1α, HIF-2α, and P4H-TM Levels Across Groups. Dots indicate extreme values.

The analysis of HIF-1α, HIF-2α, and P4H-TM levels revealed significant differences across the study groups (*p* < 0.001, for all markers). HIF-1α levels were significantly elevated in the CG/HP (+) group [7.59(5.72–9.07) ng/mL] compared to both CG/HP (−) [2.81(1.98–3.04) ng/mL] and control groups [2.31(1.54–2.72) ng/mL]. Similarly, HIF-2α levels were markedly higher in the CG/HP (+) group [17.55(16.04–20.72) ng/mL] compared to CG/HP (−) [6.84(6.31–9.12) ng/mL] and control groups [6.57(5.68–7.24) ng/mL]. P4H-TM levels followed the same pattern, with significantly higher values in the CG/HP (+) group [5.06(3.73–6.76) ng/mL] compared to CG/HP (−) [1.72(1.28–3.02) ng/mL] and control groups [1.50(1.33–2.02) ng/mL]. (Table 1, Figure 1).

The ROC curve analysis demonstrated excellent diagnostic performance for all three markers in distinguishing CG/HP+ from other groups. HIF-1 showed the highest accuracy with an AUC of 0.983 (95% CI: 0.960–1.0), achieving 100% sensitivity and 93.3% specificity at a cutoff value of 3.5. HIF-2α demonstrated similar performance (AUC: 0.981, 95% CI: 0.956–1.0) with 100% sensitivity and 91.1% specificity at a cutoff value of 10.6. P4H-TM showed good diagnostic accuracy (AUC: 0.927, 95% CI: 0.841–1.0) with 85% sensitivity and 95.6% specificity at a cutoff value of 3.5. When distinguishing CG/HP+ from the control group, both HIF-1α and HIF-2α achieved perfect diagnostic accuracy (AUC: 1.00) with 100% sensitivity and specificity (Table 2).

Correlation analysis identified several significant associations across the various groups. In the CG/HP+ group, HIF-1α exhibited positive correlations with both HIF-2α (r = 0.454; *p* = 0.044) and P4H-TM (r = 0.579; *p* = 0.007). Additionally, a significant negative correlation was observed between HIF-1α and HDL (r = −0.612; *p* = 0.004) (Table 3).

## 4. Discussion

Gastritis is a condition characterized by inflammation of the stomach lining, which may present in either acute or chronic form. While it is commonly caused by *H. pylori* infection, other factors such as alcohol use, stress, and the consumption of nonsteroidal anti-inflammatory drugs (NSAIDs) can also contribute to its development. The condition can cause symptoms such as pain, nausea, and vomiting, and if left untreated, it may lead to more severe complications like ulcers or even gastric cancer [11]. Studies have shown that 35% of cancer cases due to new infections are associated with *H. pylori* and that this bacterium has become the most common cause of carcinogenic infection worldwide [12]. Genetic and environmental factors are known to be important factors in *H. pylori* infection. However, studies related to *H. pylori* positivity and hypoxia are still limited, and this issue remains unknown. The most important finding of this study is that the serum levels of HIF-1α, HIF-2α, and P4H-TM were significantly elevated in the *H. pylori* (+) patients. Serum HIF-1α, HIF-2α, and activity were highest in the *H. pylori* (+) patients. HIF-1α, HIF-2α, and P4H-TM showed high diagnostic accuracy in distinguishing *H. pylori* (+) patients from other groups. In the *H. pylori* (+) patients, HIF-1α showed a positive correlation with both HIF-2α and P4H-TM and a negative correlation with HDL. In the *H. pylori* (−) patients, HIF-1α positively correlated with HIF-2α and folate levels. These findings suggest that HIF-1α, HIF-2α, and P4H-TM biomarkers may play a significant role in the diagnosis of *H. pylori* (+) patients and are associated with lipid profile changes.

In our study, lipid profile parameters, including total cholesterol (*p* = 0.002), triglycerides (*p* = 0.018), and LDL (*p* < 0.001), were significantly elevated in the CG patients with the CG/HP+ group compared to both *H. pylori*-negative patients and healthy controls. These findings are consistent with the results reported by Chimienti et al. [13], who observed significantly increased levels of LDL and Lipoprotein(a) in *H. pylori*-infected individuals, suggesting a potential atherogenic effect of the infection. Similarly, Rasmi et al. [14] found higher total cholesterol and triglyceride levels and lower HDL levels in *H. pylori*-positive patients with Cardiac Syndrome X. These consistent observations across different populations and disease contexts support the hypothesis that *H. pylori* infection may contribute to dyslipidemia through systemic inflammatory pathways, potentially influencing cardiovascular and metabolic risk profiles. *H. pylori* employs various molecular mechanisms to evade host immune defenses and promote chronic gastric inflammation, including modulation of cytokine responses, interference with immune cell signaling, and induction of regulatory T cells, as detailed by Myrou [15], who also discusses therapeutic strategies targeting these pathways to prevent gastric carcinogenesis and MALT lymphoma. *H. pylori* adapts to low-oxygen (hypoxic) conditions in the gastric mucosa through several key mechanisms that promote its persistence and chronic infection. These include modulation of bacterial metabolism and virulence factors to enhance survival under hypoxia, as well as activation of host inflammatory pathways that sustain a chronic inflammatory environment favorable for bacterial colonization [16]. The ability to sense and respond to hypoxia allows *H. pylori* to evade immune clearance and maintain long-term infection in humans.

HIF-1α is a key transcription factor that orchestrates cellular adaptation to low oxygen levels by promoting the expression of genes responsible for oxygen transport, energy metabolism, and the formation of new blood vessels. HIF-1α is linked to inflammation and immune responses, contributing to conditions like gastritis. HIF-2α, like HIF-1α, also responds to hypoxia but primarily regulates genes related to erythropoiesis and renal function. Myeloid-derived HIF-1α plays a protective role in *H. pylori*-induced gastritis by enhancing innate immune responses and modulating inflammation. It supports the activation of immune cells and production of pro-inflammatory cytokines essential for bacterial control [5]. While the referenced study by Skuli et al. [17] primarily focuses on endothelial HIF-2α in angiogenesis, it underscores the broader importance of HIF isoforms in regulating pathological processes, including inflammation and tissue remodeling. HIF-2α is involved in cancer and inflammation, including gastric diseases. The impact of HIF-1α activation during infections remains unclear, with uncertainty surrounding whether it has a protective or harmful effect on the host. However, myeloid HIF-1 plays a protective role in *H. pylori*-induced gastritis, highlighting the intricate balance between innate immune responses and inflammatory pathways in the development of this condition. In the present study, serum HIF-1α and HIF-2α levels were highest in the HP (+) patients. The finding that serum HIF-1α and HIF-2α levels were highest in the HP (+) patients suggests that these markers may be associated with the inflammatory response induced by *H. pylori* infection. The elevated levels of HIF-1α and HIF-2α could reflect the body’s attempt to adapt to the hypoxic conditions in the gastric mucosa caused by the infection. This could potentially play a role in regulating immune responses and tissue remodeling during the progression of gastritis. Recent studies highlight that HIF-1α plays a key role in bacterial infections, with its activation being influenced by the type of pathogen and host factors. However, it remains unclear whether HIF-1α activation is protective or harmful to the host, as its effects may vary depending on the context of the infection [9]. HIF-2 plays a significant role in the body’s response to infection and inflammation. Like HIF-1, HIF-2 is activated under low oxygen conditions, but it primarily regulates genes involved in erythropoiesis (red blood cell production) and other metabolic processes. In infections, HIF-2 can modulate immune responses and inflammation, influencing the severity and progression of diseases. Its activation may help the body adapt to the hypoxic microenvironment created during infection but may also contribute to chronic inflammation and tissue damage, depending on the context [18]. The deletion of HIF-2α in myeloid cells resulted in an increased expression of proinflammatory cytokines. Specifically, HIF-2α knockout in macrophages led to elevated levels of IL-6, a key cytokine that plays a critical role in exacerbating subsequent colon inflammation [19]. Helicobacter pylori, a bacterium that infects the stomach lining, is a major cause of chronic gastritis, peptic ulcers, and gastric cancer. It triggers an inflammatory reaction within the gastric mucosa, which contributes to tissue damage and disease progression. Moreover, *H. pylori* infection has been linked to alterations in hypoxia-related pathways, particularly those involving HIF-1α and HIF-2α. However, the specific role of HIF-2α in gastric cell survival, proliferation, or depletion during *H. pylori* infection remains poorly understood and warrants further investigation in future studies focused on its impact on gastric function [20,21,22]. HIF-1α activation during infections enhances immune responses by promoting inflammation and antimicrobial activity. Medically, modulating HIF-1α could help control excessive inflammation or improve pathogen clearance, offering potential therapeutic strategies. HIF-1α coordinates cellular adaptation to hypoxia by regulating the expression of genes involved in angiogenesis, metabolism, and survival, enabling cells to maintain function and energy balance under low oxygen conditions. Biomarkers for HIF-1α, HIF-2α, and P4H-TM show potential in distinguishing H. pylori-positive patients due to their roles in hypoxia and inflammation pathways activated during infection. As noted by Bakleh and Al Haj Zen [23], HIF-1α and HIF-2α have distinct roles in hypoxia: HIF-1α is primarily active in acute hypoxia and regulates metabolic adaptation, while HIF-2α functions in chronic hypoxia, promoting erythropoiesis and vascular remodeling. These differences may account for the differential gene expression observed in our results. However, further validation is needed to confirm their diagnostic specificity and clinical utility.

P4H-TM is an enzyme involved in the hydroxylation of proline residues in proteins, which is essential for collagen stability and the formation of extracellular matrix components [8]. It is important in fibrosis and tissue remodeling related to CG and gastric inflammation. The endoplasmic reticulum P4H-TM is a fourth variant of P4H involved in regulating HIF stability, although it may also play additional biological roles. HIF, a dimer composed of α and β subunits, along with HIF-prolyl 4-hydroxylases (HIF-P4Hs), are crucial components in the cellular response to hypoxia [6,24,25,26,27]. In the present study, serum P4H-TM levels were found to be significantly higher in the *H. Pylori* (+) patients, as were HIF-1α and HIF-2α levels. In the *H. Pylori* (+) patients, HIF-1α showed positive correlations with HIF-2α and P4H-TM. The present study demonstrates that serum P4H-TM levels, along with HIF-1α and HIF-2α levels, were significantly elevated in *H. pylori* (+) patients. This suggests a potential interaction between these markers in response to *H. pylori* infection. The positive correlations observed between HIF-1α, HIF-2α, and P4H-TM in these patients imply that these markers may work together in the inflammatory and hypoxic response induced by the infection. The findings highlight the possible role of HIF-1α, HIF-2α, and P4H-TM in modulating the immune and tissue remodeling processes in *H. pylori*-related gastritis. No data is available on the role of any HIF-P4H in *H. pylori* infection-related gastritis, but HIF has been reported to have both protective and pathological roles in this condition. Like atherosclerosis, HIF activation in *H. pylori* (+) patients may have dual effects, potentially contributing to both the immune response and tissue damage during chronic infection. P4H-TM dysfunction can impair collagen biosynthesis and proper protein folding, both critical for extracellular matrix stability. This may lead to delayed wound healing due to reduced structural support and dysregulated cell signaling. Additionally, altered hydroxylation activity can contribute to abnormal fibroblast activation, promoting excessive matrix deposition and fibrosis in tissues [8,24]. Further research is needed to clarify the specific role of HIF-P4H and HIF in *H. pylori*-induced inflammation and its long-term impact on gastric health [28]. The ROC curve analysis showed that HIF-1α, HIF-2α, and P4H-TM are highly accurate markers for distinguishing *H. pylori* (+) patients from other groups, with HIF-1 and HIF-2α demonstrating near-perfect diagnostic performance. P4H-TM also showed good accuracy. Both HIF-1α and HIF-2α achieved perfect sensitivity and specificity when distinguishing *H. pylori* (+) patients from healthy people. The perfect sensitivity and specificity of HIF-1α and HIF-2α in distinguishing CG/HP+ from the control group underscore their potential for accurate and reliable diagnosis in clinical practice. Concerns have been raised about the long-term safety of HIF-P4H inhibitors, as tumors often express high levels of HIF, which is linked to increased mortality. While HIF stabilization in tumors occurs due to intratumoral hypoxia, and HIF itself is not proven to be oncogenic, caution is needed when administering HIF-P4H inhibitors due to potential risks in cancer patients [28,29,30]. In H. pylori infection, chronic inflammation and hypoxia activate HIF-1α and HIF-2α, which modulate lipid metabolism by regulating genes involved in lipid transport and storage. P4H-TM, by controlling HIF stability, indirectly influences these pathways. Consequently, altered lipid profiles observed in infected patients may reflect HIF-driven metabolic changes. HIF-1α and HIF-2α are key regulators of the host response to hypoxia and inflammation induced by *H. pylori*. They modulate immune signaling, epithelial barrier function, and cellular metabolism during infection. P4H-TM, by hydroxylating and regulating the stability of HIFs, indirectly influences these pathogenic processes. Together, these factors may contribute to the chronic inflammation and tissue remodeling observed in *H. pylori*-associated gastric diseases.

### The Study of Limitations

This study is subject to certain limitations, the most notable being the relatively small sample size, which may restrict the broader applicability of the results. Therefore, further research involving larger and more diverse populations is recommended to confirm and extend the present findings. All participants were recruited from a single center, potentially introducing selection bias. Furthermore, the cross-sectional design prevents establishing causal relationships between hypoxia-related biomarkers and *H. pylori*-associated gastritis. Additionally, the potential causes or associated conditions of H. pylori-negative gastritis were not investigated in this study and remain unknown. This represents an important limitation, and future research should address this gap to provide a more comprehensive understanding. Longitudinal, multi-center studies are needed to confirm and extend these findings.

## 5. Conclusions

This research underscores the important role of HIF-1α, HIF-2α, and P4H-TM as biomarkers in differentiating *H. pylori* (+) patients from other groups in the study. The increased levels of these markers in *H. pylori* (+) patients indicate their potential as effective diagnostic tools, as demonstrated by the excellent performance in the ROC curve analysis. Furthermore, the lipid profile abnormalities observed in these patients highlight significant metabolic differences compared to other groups. Correlation analysis also reveals intricate relationships between these markers and various clinical parameters, including lipid levels and folate. These findings offer valuable insights into the pathophysiology of *H. pylori* infection and suggest that HIF-1α, HIF-2α, and P4H-TM could be promising biomarkers for diagnosis and possibly for tracking treatment efficacy in future studies. However, further research is needed to better understand the precise mechanisms through which these hypoxia-related markers influence the development of *H. pylori*-induced diseases.

## Figures and Tables

**Table 1:** Comparison of HIF-1α, HIF-2α, P4H-TM, and laboratory parameters among chronic gastritis, *H. pylori* (+) patients, and controls.

	Groups			
	CG/HP(+)	CG/HP(−)	Control	
	Mean ± SD or Median (25p–75p)	Mean ± SD or Median (25p–75p)	Mean ± SD or Median (25p–75p)	*p*
Age (Year)	45.95 ± 15.59	45.8 ± 17.06	45.56 ± 7.72	0.995 ^Ω^
BMI (kg/m^2^)	24.75(22.85–26.75)	25.22(24.23–26.48)	25.13(23.67–26.03)	0.675 ^¶^
ESR (1 h)	16 (7–25)	14.5 (6.5–20)	13 (10–15)	0.497 ^¶^
WBC (10^6^µ/L)	6800(5590–7845) ^a,b^	6265(5550–6900) ^a^	7700(6500–8400) ^b^	0.031 ^¶^
RBC (x 10^9^/L)	4.76 ± 0.72	4.75 ± 0.59	4.67 ± 0.58	0.870 ^Ω^
HGB (g/dL)	13.96 ± 2.18	13.46 ± 1.74	13.46 ± 1.84	0.629 ^Ω^
PLT (10^3^µ/L)	247.65 ± 64.01	257.15 ± 81.94	261.35 ± 86.42	0.685 ^†^
NEUT (10^3^µ/L)	3.9(3.3–4.9)	3.45(2.9–4.1)	3.70(2.9–4.1)	0.212 *
LYMP (10^3^µ/L)	2.32 ± 0.51	2.27 ± 0.6	2.38 ± 0.72	0.779 ^†^
EOS (10^3^/mm^3^)	0.12(0.08–0.25)	0.14(0.06–0.25)	0.13(0.06–0.25)	0.828 *
MONO (10^3^µ/L)	0.52 ± 0.19	0.55 ± 0.2	0.50 ± 0.15	0.617 ^†^
CRP (mg/L)	1.75(1–3.05)	2.25(1–4.65)	2.55(2.04–3.04)	0.248 ^¶^
T. Cholesterol (mg/dL)	212(190–242) ^a^	179(165.5–236.5) ^a,b^	176(163–186) ^b^	0.002 ^¶^
Triglyceride (mg/dL)	128.65 ± 52.95 ^a^	115.35 ± 41.65 ^a,b^	94.32 ± 21.96 ^b^	0.018 ^Ω^
HDL (mg/dL)	53.65 ± 12.75	50.45 ± 14.01	48.64 ± 7.96	0.358 ^Ω^
LDL (mg/dL)	131.5(115–157) ^a^	111(99–165) ^a^	96(83–102) ^b^	<0.001 ^¶^
AST (U/L)	20.3 ± 4.59	22.2 ± 5.72	18.8 ± 3.89	0.254 ^†^
ALT (U/L)	18.25 ± 7.68	16.85 ± 7.25	16.25 ± 6.88	0.557 ^†^
T.Protein (g/dL)	74.95(72.5–77.2)	73.95(72.35–75.6)	79.65(74.5–82.2)	0.457 *
Albumin (g/dL)	47.45 ± 3.64	46.51 ± 2.14	49.45 ± 4.44	0.325 ^†^
Iron (μg/dL)	78.5(66–122.5)	80.5(54–107)	82.25(60–112.5)	0.552 *
Iron binding capacity (μg/dL)	264.6 ± 89.91	271.9 ± 82.94	273.6 ± 90.21	0.791 ^†^
B 12 Vitamin (pg/mL)	298(239.5–387)	351.5(292.5–433.5)	376(259.5–432)	0.273 *
Ferritin (ng/mL)	50.25(21.1–105.6)	63(33–100)	60.45(28.1–100.6)	0.818 *
Folate (μg/L)	9.6(7.6–13.1)	8.8(6.65–10.85)	10.4(8.2–14.1)	0.551 *
HIF-1α (ng/mL)	7.59(5.72–9.07) ^a^	2.81(1.98–3.04) ^b^	2.31(1.54–2.72) ^b^	<0.001 ^¶^
HIF-2α (ng/mL)	17.55(16.04–20.72) ^a^	6.84(6.31–9.12) ^b^	6.57(5.68–7.24) ^b^	<0.001 ^¶^
P4H-TM (ng/mL)	5.06(3.73–6.76) ^a^	1.72(1.28–3.02) ^b^	1.5(1.33–2.02) ^b^	<0.001 ^¶^

^¶^: Kruskal–Wallis test; ^Ω^: one-way ANOVA test; ^†^: independent samples *t*-test; *: Mann–Whitney U test were applied. ^a,b^: Different superscript letters indicate significant differences. CG/HP (+): Chronic gastritis and H. pylori (+); CG/HP (−): Chronic gastritis and H. pylori (−); SD: standard deviation; 25p–75p: 25th percentile–75th percentile; *p*: *p* value; BMI: Body mass index; ESR: erythrocyte sedimentation rate; WBC: White blood cell count; RBC: Red Blood Cell (erythrocyte); HGB: Hemoglobin; PLT: Platelets; NEUT: Neutrophil; LYMP: Lymphocyte; EOS: Eosinophil; MONO: Monocyte; HDL: high-density lipoprotein cholesterol; LDL: low-density lipoprotein cholesterol; AST: aspartate aminotransferase; ALT: Alanine aminotransferase.

**Table 2:** ROC Analysis Results for Diagnostic Efficacy of HIF1, HIF2, and HTP4.

	Variable	AUC	95% CI	*p* Value	Cutoff	Sensitivity	Specificity
CG/HP (+) vs. Others	HIF-1	0.983	0.960–1.0	<0.001	3.5	100%	93.3%
	HIF-2	0.981	0.956–1.0	<0.001	10.6	100%	91.1%
	HTP-4	0.927	0.841–1.0	<0.001	3.5	85%	95.6%
CG/HP (+) vs. CG/HP (−)	HIF-1	0.963	0.910–1.0	<0.001	3.5	100%	85%
	HIF-2	0.958	0.903–1.0	<0.001	10.6	100%	80%
	HTP-4	0.895	0.791–0.999	<0.001	3.5	85%	90%
CG/HP (+) vs. Control	HIF-1	1.00	1.0–1.0	<0.001	3.5	100%	100%
	HIF-2	1.00	1.0–1.0	<0.001	10.2	100%	100%
	HTP-4	0.952	0.871–1.0	<0.001	2.3	95%	92%

Note: CG/HP (+): Chronic gastritis and *H. pylori* (+); CG/HP (−): Chronic gastritis and *H. pylori* (−); AUC: Area under curve; CI: Confidence interval.

**Table 3:** Correlation Analysis of HIF-1α, HIF-2α, P4H-TM, and laboratory parameters in groups (Spearman correlation analysis was applied).

		CG/HP(+)		
Variables	r/*p*	HIF-1 (ng/mL)	HIF-2 (ng/mL)	P4H-TM (ng/mL)
HIF-1α (ng/mL)	r		0.454 *	0.579 **
	*p*		0.044	0.007
HIF-2α (ng/mL)	r			0.444 *
	*p*			0.05
HDL (mg/dL)	r	−0.612 **	−0.284	−0.275
	*p*	0.004	0.225	0.241

Note: CG/HP (+): Chronic gastritis and *H. pylori* (+); r: Correlation coefficient; *p*: *p* value. *: *p* < 0.05; **: *p* < 0.01.

## Data Availability

The datasets used and/or analyzed during the current study are available from the corresponding author on reasonable request.
